# A social-healthcare pathway to facilitate return to work of cancer survivors in Italy: The UNAMANO project

**DOI:** 10.3233/WOR-205249

**Published:** 2021-12-20

**Authors:** Sara Paltrinieri, Elena Ricchi, Elisa Mazzini, Elena Cervi, Elisa Sandri, Stefania Fugazzaro, Stefania Costi

**Affiliations:** a Physical Medicine and Rehabilitation Unit, Azienda Unità Sanitaria Locale-IRCCS di Reggio Emilia, Italy; b Medical Directorate, Azienda Unità Sanitaria Locale-IRCCS di Reggio Emilia, Italy; c In-Forma Salute, Medical Library, Azienda Unità Sanitaria Locale-IRCCS di Reggio Emilia, Italy; d Department of Surgery, Medicine, Dentistry and Morphological Sciences, Università di Modena e Reggio Emilia, Italy; e Scientific Directorate, Azienda Unità Sanitaria Locale-IRCCS di Reggio Emilia, Italy

**Keywords:** Neoplasms, occupational therapy, comprehensive health care

## Abstract

**BACKGROUND:**

Return to work (RTW) is a major goal to promote cancer survivors’ social participation.

**OBJECTIVE:**

This study describes the multidisciplinary social-healthcare pathway called UNAMANO, conceived to support RTW in this population.

**METHODS:**

UNAMANO was developed by the Azienda USL-IRCCS di Reggio Emilia, in partnership with the local branch of the Italian Medical Association, nonprofit associations, vocational training institutions, social cooperatives, a labour union, and a chartered accounting firm.

**RESULTS:**

UNAMANO is directed towards employed individuals diagnosed with cancer living in the province of RE. It was developed through four actions: A) training healthcare professionals on work-related occupational rehabilitation; B) dissemination among community and stakeholders; C) recruitment and training of volunteers; D) cancer survivor engagement and provision of a personalized comprehensive intervention. This consists in providing information and either occupational therapy to overcome barriers and facilitate RTW or social support through re-training and tailored job search strategies based on individual risk of job loss.

**CONCLUSIONS:**

UNAMANO is the first Italian multidisciplinary social-healthcare pathway supporting RTW of cancer survivors. Addressing a wide area of cancer survivors’ needs, it provides personalized intervention to resolve work-related issues. We propose this patient-centred RTW model to promote an easier transition from hospital to community.

## List of abbreviations

RTW: Return to work

CSs: Cancer survivors

RE: Reggio Emilia

OECI: Organization of European Cancer Institute

WelCom: Community Welfare

OTs: Occupational Therapists

OQ: Occupational Questionnaire

## 1 Background

The number of individuals living with or beyond cancer grows every year due to the increasing incidence of cancer diagnosis, thanks in part to early diagnosis, and to the improved effectiveness of treatments [1]. In 2018, new cancer diagnoses in Europe reached almost four million [2], representing 23% of new diagnoses worldwide [2, 3]. Moreover, one-third of new diagnoses regarded individuals in their working age [4] who might have needed to return to work (RTW) during or after cancer treatment. Thus, RTW is a major goal in order to promote cancer survivors’ (CSs) social participation, which corresponds to fully carrying out activities of daily living and social roles [5]. Employment is key to determining an individual’s social participation [6–8] since it is an essential element of self-identity and self-esteem, it provides financial support to the individual and his/her family, it allows one to express his/her abilities and talents, it gives normalcy to daily structure and, finally, it results in better health [9, 10]. Moreover, RTW provides the opportunity to engage socially with colleagues and other individuals [11].

In the late 20th century, RTW of CSs is estimated to be 64% in the USA and in Europe (range 30–94%) [12], with a variety of factors negatively influencing it [13]. Unemployment risk is 1.48 times higher in CSs than in healthy controls [14]. For instance, those CSs who perceive lower work performance and work satisfaction retire early, as do individuals with physically demanding work [15]. Moreover, unsupportive work environments act as a barrier to the RTW process [16, 17]. Finally, the type of cancer and the treatment-related side effects have negative repercussions on employment overall [18–20].

RTW of CSs is also an indicator of rehabilitation success [21], even though the work-related consequences of cancer are not always addressed in cancer recovery [22, 23]. As a matter of fact, RTW of CSs seems to be a neglected topic in different areas of Europe, as highlighted by the literature [21, 24]. Nowadays, few interventions exist that help people with cancer maintain their engagement and job performance at work [22]. These interventions would also widely support social participation through recovery in the physical, cognitive, emotional, and interpersonal domains of functioning, which may have declined during cancer treatment [25]. To date, multidisciplinary approaches including a combination of physical exercises, patient education, counselling, behavioural training and/or vocational counselling have shown a moderate level of effectiveness in promoting RTW for individuals with cancer and other chronic conditions [26–29]. To be effective, these approaches need to be personalized and adapted to the social and organizational environment [27, 28]. For these reasons, we considered it appropriate to collect contextualized data on this topic and by developing suitable rehabilitation aimed at the early promotion of RTW of CSs. We therefore developed a multidisciplinary social-healthcare pathway we called UNAMANO, which is the Italian translation of “one hand” and has the meaning of offering support. The feasibility of UNAMANO is currently being tested, and we describe it in detail in this study so that it can be adapted to and replicated in similar contexts.

## 2 Methods

### 2.1 The rationale

This research project started in 2014 in response to a recommendation received from the Accreditation and Designation Programme of the Organization of European Cancer Institutes (OECI). UNAMANO is the main action implemented by the Azienda USL-IRCCS di Reggio Emilia (Italy) to facilitate the social participation of CSs. Firstly, a systematic review of the European literature was conducted to collect up-to-date data regarding RTW of CSs [24]. That review focused on the RTW rate of CSs employed at the time of diagnosis, their sick leave pattern, and on factors that acted as facilitators of or barriers to the process of work reintegration. It also highlighted the almost complete absence of data collected on this topic in southern and central Europe. Meanwhile, a cross-sectional survey was carried out to collect contextualized information about the RTW process of CSs living in the province of Reggio Emilia (RE) [30]. Although characterized by good prognosis and employed at the time of diagnosis, half of CSs reported difficulties in the RTW process, attributable to the social environment at work, work tasks, health status, and patients’ perspective regarding their work ability.

### 2.2 The context

This pathway was developed in the province of RE, Emilia-Romagna Region, in northern Italy. This province covers an area of 2,291 km^2^, accounts for a population of 530,000 and includes one medium-sized city (Reggio Emilia –population 167,000) and numerous smaller towns and villages. The large main hospital is in the city of Reggio Emilia and five smaller provincial hospitals are in each of the five health districts. Cancer treatment is provided in the main hospital as well as in three of the provincial hospitals.

In 2014, cancer incidence (excluding non-mela-noma skin cancer) in the province of RE reached 3,205 cases (0.6% of the population). The most common cancer diagnoses were lung and prostate cancer among men (both 15%) and breast cancer (30%) among women; the 5-year survival rate of all cancer sites was 62.1% [31]. According to Italian Law 118/1971, the majority of patients with cancer are legally recognized as invalid for the duration of treatment and can thus receive sick pay, but only for a limited number of months, unless the disease results in significant and permanent functional impairment [32].

Thus, the majority of CSs do not benefit from medium-long-term financial and legislative protection. CSs are therefore potentially vulnerable [33] as they might need to return to work soon after diagnosis, even in the presence of treatment-related side effects, to maintain their financial independence.

There are nearly twenty non-profit volunteer associations in the province that offer different services to cancer patients and to their caregivers, e.g. home health assistance, fundraising, wig banks, and so on. Thus, the local social context is clearly very aware of and sensitive to cancer and the cancer-related consequences that might impact on the everyday life of diagnosed patients and their families.

The local economy is based on different sectors: agriculture and animal farming as well as widespread, specialized industry, including the ceramic industry, construction materials and technologies, mechanical engineering, the automotive industry, the food processing and agroindustry, biomedical industries, fashion and textiles, and tourism [34, 35]. According to the latest data from the Italian National Institute of Statistics (Istat), in 2016 there were 41,505 active companies in the province of RE, 58% of which registered as self-employment [36]. Most of the active companies (94%) had fewer than 10 employees, 5% had 10 to 49 employees, and only 1% had more than 50 employees. In the same year, the employment and the unemployment rates for individuals from ages 15 to 64 years were 68.2% and 4.7%, respectively [36]; the latter was considerably lower than the Italian average. Even though the province of RE is one the major economic areas in Italy and in Europe [37], the economic crisis of the last decade may be responsible for the increase in vulnerability and in poverty, which account for 30% and 3% of the population, respectively [38]. Thus, in 2016 the Community Welfare (WelCom) initiative was launched by the local Manodori Foundation with the purposes of detecting the local socioeconomic vulnerability and of planning innovative services aimed at preventing a dangerous downward spiral into poverty by supporting individuals with difficulties. WelCom sponsored UNAMANO, which was developed over the course of seven workshops held between January 2017 and July 2017. A project manager facilitated the cooperation between several local public and private companies and institutions that endorsed the project, each with a different area of expertise. Altogether, they created the specific network of UNAMANO, which includes the Local Health Authority, the local branch of the Italian Medical Association, four non-profit volunteer associations, three vocational training institutions, four social cooperatives, a labour union, and a chartered accounting firm (see Appendix A for a detailed list). Since it was launched in November 2017, UNAMANO has been providing comprehensive care to address the issues encountered by CSs in the RTW process.

### 2.3 The aims of UNAMANO

The primary aim of UNAMANO is to support CSs in their RTW process by providing a personalized care intervention delivered through this new, contextualized, social-healthcare pathway. The secondary aim is to direct CSs who are at high risk of losing their job or who have already lost it to the appropriate social support.

### 2.4 Study population and eligibility criteria

UNAMANO is for all employed individuals living in the province of RE who receive a diagnosis of cancer.

### 2.5 Ethics approval

This project was approved by the Ethics Committee of the Province of Reggio Emilia (2018/8410). The study was registered on ClinicalTrials.gov under identifier NCT03666936.

## 3 Results

### 3.1 The development process of UNAMANO

UNAMANO was developed through four actions (A, B, C, D): actions A-C concern its promotion; action D consists in engaging CSs and in providing a personalized, comprehensive intervention. Action D is managed by occupational therapists (OTs) and involves several professionals who collaborate in planning a multidisciplinary intervention tailored to that CS’s specific needs. It consists of three types of support that are provided singly or in combination: information regarding employment law; occupational therapy to overcome barriers and facilitate RTW for CSs; social support through the provision of re-training and job search strategies for those CSs at high risk of job loss and for those who have unfortunately already lost their job by the time they join UNAMANO. Table 1 describes the role played by the network in each action.

**Table 1:** Actions implemented

Action	Aim	Institutions involved	Brief description
A. Healthcare professional training on work-related occupational rehabilitation	To inform all the healthcare professionals involved in the care of cancer patients about UNAMANO	•Local Health Authority	•Training events for healthcare professionals involved in cancer patient care
		•Italian Medical Association
		•One nonprofit volunteer association
B. Dissemination in the community and to the stakeholders	To raise awareness in the community and among the stakeholders	•Local Health Authority	•Creation of flyers and the website
		•Two nonprofit volunteer associations •Labour union	•Dissemination during community events
		•Dissemination through newspapers and social media
			•Brief meetings with professional and industry associations
C. Recruitment and training of volunteers	To recruit volunteers to support the initiative	•Local Health Authority	•Brief meetings with the nonprofit associations •Training events for volunteers
		•Two nonprofit volunteer associations
		•Social cooperatives
		•Vocational training institutions
		•Labour union
D. CS engagement and provision of personalized care	To engage eligible CSs and provide them with multidisciplinary, comprehensive, personalized care	•Local Health Authority •Social cooperatives •Vocational training institutions •Labour union	•CS engagement and screening
			•In-depth assessment of individuals included
			•Provision of information, occupational therapy, and social support, as needed

CSs = cancer survivors.

#### 3.1.1 Action A –Healthcare professional training on work-related occupational rehabilitation

The first action consisted in holding meetings to inform all the healthcare professionals involved in the care of cancer patients about UNAMANO. This included professionals from several disciplines working in different wards (oncology, haematology, rehabilitation, radiotherapy, and surgery). Training events were also held for the general practitioners and the occupational physicians working in the province. Training events were conducted by the healthcare professionals of the pathway, namely the OTs, three physicians, a physiotherapist, a psychologist, and a nurse. During the meetings, updated information about work reintegration was reported, the rationale of UNAMANO was illustrated, and straightforward indications for referral were provided.

#### 3.1.2 Action B –Dissemination in the community and to the stakeholders

Action B consisted in raising awareness of and providing information to the local community regarding UNAMANO. This was accomplished by such actions as creating and distributing flyers, launching the website, and publishing news items in local and national newspapers and on social media. The flyers, distributed in the hospitals throughout the province, reported the objectives of UNAMANO as well as the different types of support provided and the first contact information. The flyers were checked and reviewed for comprehension by a health literacy expert and by three CS volunteers. The flyers have recently been translated into English in order to inform other cultures in the community or other research groups about UNAMANO (Appendix B). Further, a specific project website was created and recently translated into English (www.una-mano.webnode.it); in addition to information on the project and its background, the website posts updates and news. Information on UNAMANO was also disseminated among stakeholders through local and national newspapers and in magazines that concern the healthcare and labour market sectors. Lastly, the whole UNAMANO project was shared with several local professional and industry associations representing employers’ and employees’ interests.

#### 3.1.3 Action C –Recruitment and training of volunteers

This aim of this action, started in February 2018, was to recruit volunteers to support the initiative. UNAMANO contacted several nonprofit cancer volunteer associations to describe its pathway and to identify possible areas of volunteer work. The main area identified was providing CSs with general information regarding UNAMANO and employment law. Consequently, three specific training events were organized to recruit volunteers willing to support UNAMANO. During these events (which lasted three to four hours) the OTs, the physiotherapist, and the psychologist introduced UNAMANO and talked about useful communication techniques when dealing with CSs. The Local Health Authority’s data privacy office described to the volunteers how to collect, record, and protect sensitive data and the CSs’ written informed consent. Finally, the labour union, the social cooperatives, and the vocational training institutions participating in UNAMANO explained their specific roles in the network and provided in-depth information about employment law.

#### 3.1.4 Action D –CS engagement and provision of personalized care

**Fig. 1:**
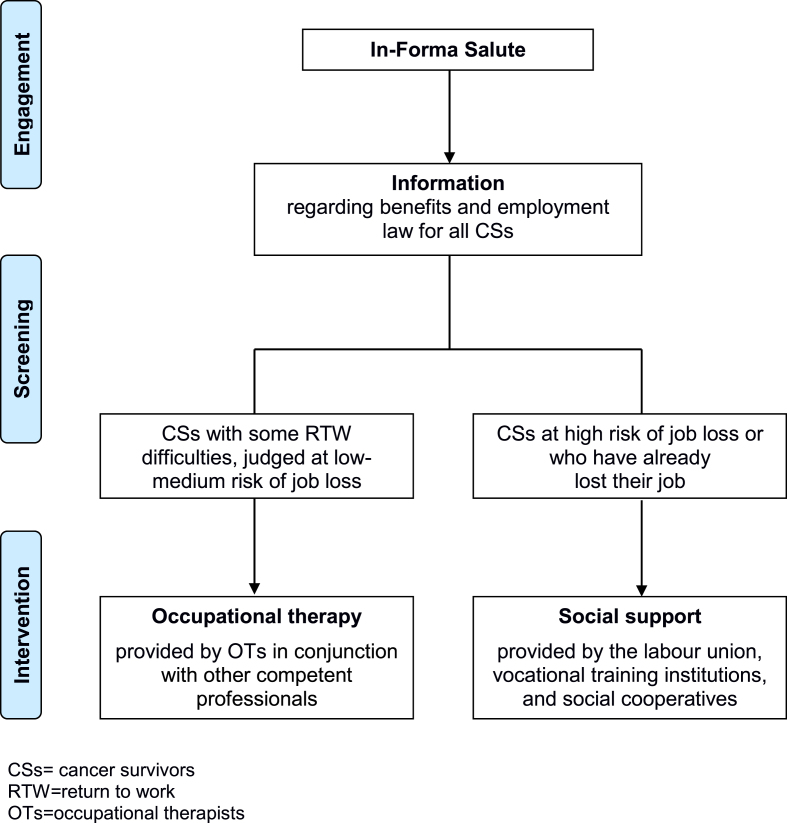
Flowchart of UNAMANO project.

This ongoing action is made up of two activities: the first is the engagement and screening of eligible individuals; the second is the provision of the multidisciplinary, personalized, comprehensive intervention (Fig. 1).

*3.1.4.1. CS engagement and screening:* In-Forma Salute, located in the main hospital of RE, is a service provided by the Medical and Patient Library of the Local Health Authority and is the point of contact with UNAMANO. The service was born in 2005 to help patients, families, and citizens obtain comprehensible, evidence-based health information. The nurse, who is also an information specialist and is responsible for this service, provides a wide range of information in response to various requests from patients and family members (e.g. health-related, logistical, etc.): a frequent patient request regards legal protection in the workplace based on employment law. For these reasons, In-Forma Salute was chosen as the project’s point of contact; citizens can access In-Forma Salute directly either in person, by telephone, or by email in order to receive personalized information. If the eligible criteria are met, the information specialist-nurse, with the volunteers’ assistance, provides information about UNAMANO and collects the written informed consent to be included in the project. Then the OTs make an appointment and proceed with the first screening, which consists in administering the Occupational Questionnaire (OQ) to define the risk of job loss (Appendix C). The OQ was developed by our research group by taking into consideration the key factors that emerged from studies conducted in Europe and locally [24, 30]. The OQ is composed of 23 closed- and 3 open-ended questions; as some sections are mutually exclusive, each participant answers an average of 13 questions. The collected information was:

– sociodemographic factors (age, education level, marital status, and family members);
– work-related factors (employed/not employed; employed in private/public sector; description of a typical workday, employment contract and classification, work schedule flexibility and flexibility in how and when to carry out daily tasks, number of colleagues, and years of employment in the current company).

The open-ended questions focus on the CSs’ perspective regarding any work-related difficulties that may be encountered during the RTW process. Questions regarding health-related factors are not included in the OQ, so recruited CSs are not required to report their cancer diagnosis and treatment course. If CSs are no longer employed, the OTs obtain more information regarding social status, employment history, and professional training with a specific Social and Work Questionnaire developed by a cooperative of the network (Appendix D).

After the first screening, recruited CSs are contacted by telephone within 10 days by the OTs for a second meeting in order to share the personalized intervention developed to overcome work difficulties.

*3.1.4.2. Provision of personalized comprehensive intervention:* Based on the information collected by the questionnaire/s, the OTs share with the individual the most suitable personalized intervention and implement it; based on the individual’s needs, other competent professionals of the pathway are involved. Therefore, CSs can receive any of the three types of support, or a combination of these: information, occupational therapy, and/or social support.

*3.1.4.2.1. Information on employment law*. Information is provided by OTs in cooperation with the information specialist-nurse and the labour union. It is delivered at the In-Forma Salute office in a face-to-face meeting. The aim is to provide information about the benefits and the legal protection in the workplace under Italian Law [32, 39, 40]. Legally recognized disability is the essential prerequisite for accessing welfare and workplace benefits: no benefits are granted to individuals with up to 33% disability; non-economic benefits are granted from 34% upwards (e.g. paid sick leave), and economic benefits may be granted from 74% upwards (e.g. disability pension). Also, from 46% disability, unemployed or part-time employees can register in the Provincial Labour Lists to find a more suitable job. Moreover, the Italian law requires companies to hire a proportion of disabled workers (at least 60% disability) based on the overall number of employees. Disabled workers are assigned to tasks compatible with their current health status and can benefit from work schedule reduction. Finally, whenever possible, disabled workers can choose between different places of work and paid leave of absence. However, for most CSs, these laws do not all apply for long as, in most cases, disability is temporary and not very severe.

*3.1.4.2.2. Occupational therapy*. Occupational therapy is guaranteed for CSs who, after the initial screening, are considered at low-medium risk of job loss or with RTW difficulties. During the second meeting, after an in-depth description of work tasks, work schedule, work environment, and the relationship with the employer and/or colleagues, the OTs, together with the CS, develop a personalized intervention, which is based on the individual’s priorities. The OTs also ask the CS for permission to involve his/her employer and occupational physician in creating a RTW plan shared by both sides (employee and employer). This is the desirable scenario: OTs evaluate the workplace location and setting and observe the CS during the execution of routine tasks. Difficulties are analysed, and the occupational therapy aimed to overcome these difficulties is implemented with the collaboration of both sides. This can include one or several of the following: accommodation of the workplace environment, accommodation of the tasks, indication to use custom disability devices, energy conservation techniques, implementation of an effective communication plan between employer, employee, and colleagues. Furthermore, based on the OTs’ assessment, further professional support from the psychologist, the labour union, the social cooperatives, and the vocational training institutions can be activated. The psychologist is involved when the OTs recognize the CS’s need to develop self-planning skills. The labour union is involved to support the individual with employment rights, when necessary mediating with the employer regarding critical issues in the RTW process (e.g. work schedule). Social cooperatives and vocational training institutions can be involved when a CS requires re-training to maintain employment at his/her current company by performing other tasks. The OTs are responsible for choosing which professional to involve, based on the assessment carried out.

If the CS does not agree to involve his/her employer and occupational physician, the OTs collect as much detailed information as possible regarding work-related factors during a face-to-face meeting or by telephone. If necessary and if possible, work-related tasks can be simulated during the meeting to allow for a more reliable description of tasks. Based on these data, the intervention is planned by the OTs and delivered to the individual. However, as the employer is not directly involved in making any accommodation suggested, the CS him/herself has full responsibility for doing so. In these cases, which are not ideal but which we expect may occur, the interventions are limited in their strategies but could still include a certain degree of accommodation of the workplace and/or tasks, the use of small disability devices, and energy conservation techniques. The individual can also be trained in more effective communication strategies to be applied in the workplace.

*3.1.4.2.3. Social support*. Social support is guaranteed to CSs who, after the initial screening, are considered at high risk of job loss and for those who, unfortunately, already lost their job before joining UNAMANO. Social support is provided by the social cooperatives, the vocational training institutions, and the labour union. The OTs’ role is to create a link between CSs and these components of the network, which will contact the individual to make an appointment at their local offices. Social support is realized through skills analysis, job search workshops, professional internships, and soft skills training. For those individuals at high risk of job loss, the labour union mediates with employers by handling the critical issues in order to facilitate a reintegration in the same company, including considering different roles or responsibilities. For those in search of a new job, the social cooperatives and the vocational training institutions help in the job search and provide training in areas that need workers. Furthermore, in response to specific needs, professional internships based on CSs’ aptitude and skills could be undertaken.

### 3.2 Data analyses

UNAMANO is a new project and is not yet in-cluded in the standard care routinely provided to CSs by the Local Health Authority. For this reason, it is extremely difficult to estimate the size of the potential target population of UNAMANO. Based on data from the Reggio Emilia Cancer Registry and on those collected in the previous cross-sectional study [30], we estimate that, every year, a minimum of 300 individuals diagnosed with cancer (excluding non-melanoma skin cancer) may wish or need to return to work, even while undergoing treatment. Fifty percent of these individuals might encounter difficulties in the RTW process. Since at this stage the project is not yet well known, and our priority is to disseminate it among the stakeholders, it is likely that a fair share of potential beneficiaries may not be engaged. For this reason, we estimated recruiting a sample of 30 CSs per year.

### 3.3 Feasibility indicators

As UNAMANO is a new project, collecting indicators to test its feasibility in this specific context of application is of utmost importance. We have identified several feasibility indicators, corresponding to each action, that have been routinely collected since UNAMANO recruitment began in May 2018:

1 Action A:
  – number of healthcare providers directly informed about UNAMANO.
  – number of healthcare professionals who refer patients to UNAMANO.
2 Action B:
  – number and type of dissemination activities implemented
  – number of individuals that contact In-Forma Salute to seek information regarding UNAMANO without being referred by a healthcare professional.
3 Action C:
  – Number of volunteers informed about UNAMANO
  – Number of volunteers recruited by UNAMANO
4 Action D:
  – number of CSs engaged in UNAMANO compared to those estimated per year.
  – number and type of personalized comprehensive interventions provided (information, occupational therapy, social support) and, for those who have been offered the involvement of the employer, the number of those who have accepted this type of intervention.
  – duration of the support provided

In order to gain insight into the effectiveness of the social-healthcare pathway planned, we also have collected data about:

– ratio of CSs who have returned to work/not returned to work among those who received only information support;
– ratio of CSs who have returned to work/not returned to work among those who received information support and occupational therapy;
– difficulties faced by CSs in the RTW process, which are collected by the OQ during the first screening and analysed qualitatively, categorizing them into emerging themes

These data will be disseminated through publication in databases or scientific journals.

## 4 Discussion

The aim of this manuscript was to describe the development process of UNAMANO, a multidisciplinary social-healthcare pathway implemented in the province of Reggio Emilia. UNAMANO has been promoted through the training of healthcare professionals, dissemination among community and stakeholders, and recruitment and training of volunteers, and consists in the provision of comprehensive personalized care for working age CSs. UNAMANO helps CSs at low-medium risk of job loss in overcoming difficulties in the RTW process and supports CSs at high risk of job loss in searching for new job opportunities.

### 4.1 Strengths of the study

This study describes the first Italian RTW care model for CSs, based on previous sound European and local epidemiological studies [24, 30] and on the features of the local context. UNAMANO was created thanks to the contribution and expertise of different professionals in the social and healthcare areas of practice. These professionals make up the network (Appendix A); working in collaboration, they implement the innovative pathway that provides broad support to CSs. In this pathway, a pivotal role is played by the OTs, who provide CSs with healthcare support aimed at improving work ability through personalized accommodations and strategies [41]. The extensive cooperation within the network, especially from those members that provide social support, allows for an exhaustive CS assessment and, consequently, comprehensive rehabilitation support. Moreover, UNAMANO has informed the main local professional and industry associations representing different economic sectors about this pathway and has gathered their perspective as significant stakeholders [42]. Integrating UNAMANO into standard cancer care practice would guarantee a prompt CS needs analysis and the consequent provision of personalized care. Thus, the transition from hospital to workplace would be facilitated and the quality of RTW would improve. The components of this RTW care model have been described in detail, as has the context of application, with the aim to provide all the necessary information to replicate it in other similar contexts. Furthermore, a wide set of feasibility indicators has been proposed and will be reported at the end of the project in order to collect data on its suitability.

### 4.2 Limitations of the study

While UNAMANO is an innovative multidisciplinary care model, implementing it in clinical practice may prove difficult [43]. In this specific context, healthcare professionals do not usually view workplace reintegration as an aspect that completes treatment [44–46]. Healthcare professionals, even those trained in work-related occupational rehabilitation, might not investigate employment status or might underestimate work difficulties and disease-related issues. Therefore, they may not refer CSs to UNAMANO. On the other hand, some CSs may not contact In-Forma Salute despite their healthcare professionals’ advice [47]. Another potential limit is that informed but ineligible individuals may contact UNAMANO for help in finding a job or in changing their current one. This might be particularly true due to the recent economic crisis, which has led to greater unemployment even in this highly industrialized area [36]. In those cases, general information support will also be provided in order to facilitate access to appropriate services. However, there is the risk that this new project is mistaken for an employment agency or service. Indeed, UNAMANO does not duplicate services already in place in the province; its aim is to fill a gap in fostering a seamless transition from hospital to workplace by linking all services in the pathway [48]. These two limits, particularly the former, could delay project implementation and its success in terms of feasibility. This is a pivotal aspect to consider because future financial support of UNAMANO will be determined by its feasibility and effectiveness and in light of other emerging priorities.

## 5 Conclusions

UNAMANO could have a positive impact on CSs’ health and well-being and it can be provided at any stage of cancer disease and treatment. UNAMANO follows CSs over time, ensuring that the CS’s work situation is in line with his/her health status and expectations.

Thanks to the OTs’ support and to specific training, healthcare professionals involved in the care of cancer patients will increase their knowledge of and be more sensitive to RTW issues. This will probably lead to the adoption of a more holistic approach to patient needs. A successful RTW could also reduce the risk of economic frailty of these patients and their families [49] and lead to a reduced, more appropriate use of healthcare services by CSs [50]. Therefore, in light of all the topics here discussed, UNAMANO could fill an important gap in the care of cancer patients.

## Supplementary Material

Supplementary Material

## Reporting standards

TIDIeR checklist has been used to describe in detail the development of UNAMANO with the aim to allow its replicability and adaptation to other contexts. The completed TIDIeR checklist is provided as additional file (Appendix E).
