# Supplementary material for: A social-healthcare pathway to facilitate return to work of cancer survivors in Italy: The UNAMANO project
Source: Work. 2021 Dec 20;70(4):1243–53. doi: 10.3233/WOR-205249 (PMC8764592; doi:10.3233/WOR-205249)
Supplement: Supplementary Material [file wor-70-wor205249-s001.pdf]

Appendix A. The UNAMANO Network

- Associazione DarVoce, Reggio Emilia
- Associazione Onlus Centro Sociale Papa Giovanni XXIII, Reggio Emilia
- Azienda Unità Sanitaria Locale-IRCCS, Reggio Emilia
- BFMR & Partners, Reggio Emilia
- Cooperativa Sociale Altra Tensione, Reggio Emilia
- Cooperativa Sociale Il Ginepro, Castelnovo né Monti
- Cooperativa Sociale Koiné, Reggio Emilia
- Fondazione ENAIP Don Magnani, Reggio Emilia
- Fondazione GRADE onlus, Reggio Emilia
- LILT, Reggio Emilia
- Ordine dei Medici e Chirurghi, Reggio Emilia
- Società Medica Lazzaro Spallanzani, Reggio Emilia
- Ust CISL Emilia Centrale e Anteas Servizi, Reggio Emilia
- Università degli studi di Modena e Reggio Emilia
- Winner Mestieri Emilia-Romagna s.c.s., Reggio Emilia

In-Forma Salute

Representative: Elena Cervi

The In-Forma Salute representative is at your disposal to provide the personalised information required and put patients in contact with professionals.

Contacts

Tel. 0522 296497 - e-mail informasalute@ausl.re.it
website www.una-mano.webnode.it

Where we are

CORE (Reggio Emilia Oncology and Haematology Centre)
In-Forma Salute, 1st floor
Main entrance CORE from via Benedetto Croce
Entrance from Arcispedale Santa Maria Nuova, via Risorgimento n. 80, 1st floor, brown path no. 8

Openings Hours

Monday to Friday from 9.00 to 13.00
Wednesday from 14.30 to 16.30

"UNA MANO, Sostegno di pazienti al lavoro" was funded by **Fondazione Manodori** di Reggio Emilia and by the healthcare authority Azienda USL-IRCCS di Reggio Emilia.

UNA MANO was performed as part of the **Welcom project** in the planning workshop "*Occuparsi di lavoro, il lavoro come occasione per intercettare nuove fragilità*" (Dealing with work, work as an opportunity for intercepting new fragilities) in association with:

- Associazione DarVoce, Reggio Emilia
- Associazione Onlus Centro Sociale Papa Giovanni XXIII, Reggio Emilia
- Azienda Unità Sanitaria Locale-IRCCS, Reggio Emilia
- BFMR & Partners, Reggio Emilia
- Cooperativa Sociale Altra Tensione, Reggio Emilia
- Cooperativa Sociale Il Ginepro, Castelnovo né Monti
- Cooperativa Sociale Koiné, Reggio Emilia
- Fondazione ENAIP Don Magnani, Reggio Emilia
- Fondazione GRADE onlus, Reggio Emilia
- LILT, Reggio Emilia
- Ordine dei Medici e Chirurghi, Reggio Emilia
- Società Medica Lazzaro Spallanzani, Reggio Emilia
- Ust CISL Emilia Centrale e Anteas Servizi, Reggio Emilia
- Università degli studi di Modena e Reggio Emilia
- Winner Mestieri Emilia Romagna s.c.s., Reggio Emilia

SERVIZIO SANITARIO REGIONALE
EMILIA-ROMAGNA
Azienda Unità Sanitaria Locale di Reggio Emilia
IRCCS Istituto in tecnologie avanzate e modelli assistenziali in oncologia

FONDAZIONE
CASSA DI RISPARMIO
DI REGGIO EMILIA
PIETRO MANODORI

UNA mano

SOSTEGNO DI PAZIENTI AL LAVORO

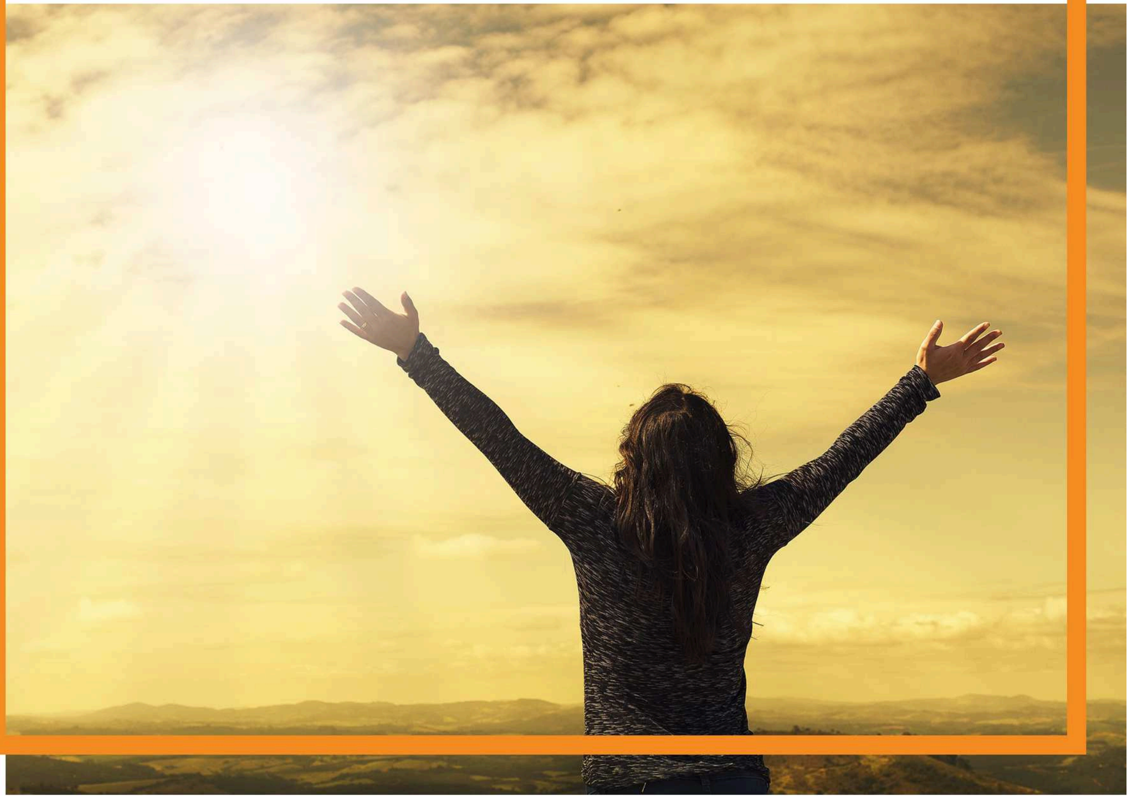

WHAT IS "UNA MANO, SOSTEGNO DI PAZIENTI AL LAVORO" *(One hand, support for patients at work)*

"UNA MANO" was created to help workers with cancer to keep their jobs and return to work.

We are aware that many people diagnosed with cancer are in the working age group, therefore they need to work during and after their treatment plan.

People diagnosed with cancer could experience changes that make returning to work problematic and not very pleasant.

To better help them return to work, it is important to focus on different aspects including the characteristics of the person, the work environment and the disease. For this reason, a health and social rehabilitation intervention to help people with cancer to keep their jobs and return to work is a fundamental part of this kind of support.

EXPERIENCES OF PATIENTS INTERVIEWED: EXAMPLES OF DIFFICULT WORK SITUATIONS

"When I went back to work…
…I had trouble getting used to the pace of work"
N. age 45 (Secretary)

"After a period of absence from work due to illness I realised there was a gap between my IT knowledge and that of my colleagues, but I wasn't given the chance to get up to date"
S. age 51 (Web master)

"…Standing up for a long time and lifting heavy loads made me really tired"
T. age 26 (Warehouse operator)

"…I was less attentive and precise while taking orders at the table, and I also gave out the wrong change…"
D. age 42 (Pizza waitress)

"Writing on the board made my arm numb and tired…"
R. age 48 (Teacher)

WHO IS IT FOR

"UNA MANO" is for all those who have been diagnosed with cancer and who think they will have or are experiencing problems in the workplace.

WHAT WE OFFER

"UNA MANO, Sostegno di pazienti al lavoro" aims to help people with cancer to overcome the problems that they are experiencing in the workplace.

Those who are having problems in the work environment can:

- Meet a professional occupational therapist to identify the problem at work and agree on the objectives to be reached
- Receive informative support for facing the perceived difficulty in the workplace
- Receive a social security and care consultation
- Agree on the healthcare and/or social support in the workplace
- Receive healthcare and/or social support through a personalised rehabilitation intervention
- Perform work placements
- Perform a cross-discipline skills training and analysis plan
- Perform an individual orientation plan
- Receive information on possible financial aid (in the event of debt), according to the provisions of Italian Law 3/2012.

You can contact the In-Forma Salute representative who will answer your questions with correct and up-to-date information and, according to the situation, put you in contact with the **Occupational Therapist** for an initial assessment.

To help you to keep your job and return to work, the Occupational Therapist and the social-healthcare pathway professionals will organise an intervention that considers the requirements and the objectives that the person wishes to achieve.

Appendix C. Occupational Questionnaire

Nurse, OT, or volunteer:…………………………………………..

Date: …………………

1) Cancer survivor's request:

………
………
………
………
……………………

2) Was the CS referred to the UNAMANO project?

|_|Yes (go to question 3 and then to the Occupational Questionnaire (p.2))

|_| No (provide information about In-Forma Salute contacts and timetable.

3) Who referred the CS to In-Forma Salute?

|_| General Practitioner

|_| Specialist

(Indicate whether an oncologist, haematologist, physiatrist, etc.)

…………………………………………………….

|_| Other healthcare professional

(Indicate whether a nurse, physiotherapist, psychologist, etc.)

…………………………………………………….

|_| Occupational Physician

|_| Nonprofit volunteer association

|_| Other: …………………………………………………

OCCUPATIONAL QUESTIONNAIRE

Section A_ Personal Information

Name and Surname: ……………………………………………………

Age: ……………………………………………………

Contacts (telephone number, email address):……………………………………………………

Address;……………………………………………………

A1. Marital Status:

|_| Married/Cohabitant

|_| Unmarried

|_| Separated/Divorced

|_| Widowed

A2. Number of children: /______/

A3. Education level:

|_| None

|_| Elementary school

|_| Middle school

|_| High school diploma

|_| University degree

|_| Other……………………………………………

A4. At the moment you are:

1|_| Unemployed (if unemployed, go to section B)

2|_| Employed (if employed, go to section C)

Section B_ Unemployed

B1. At your last job, what was your position?

|_| Executive

|_| Manager

|_| Office Worker

|_| Worker

|_| Teacher

|_| Food service worker

|_| Homemaker

|_| Other ……………………………………………………..

B2. Do you do any volunteer work?

|_| Yes

|_| No

B3. Why have you come to In-Forma Salute? What is your work-related problem?

………
………
………
………
……………………

Section C_ Employed

C1. You work in the:

|_| Public sector (go to section D)

|_| Private sector (go to section E)

Section D_ Employed in the public sector

D1. You are:

|_| Self-employed

|_| Employed

D2. If you are employed, you have a:

|_| Fixed-term contract

|_| Permanent contract

D3. You work as:

|_| Executive

|_| Manager

|_| Office Worker

|_| Worker

|_| Teacher

|_| Food service worker

|_| Homemaker

|_| Other …………………………………………………..

D4. Does your job have a flexible schedule?

|_| Yes

|_| No

D5. Does your job have work-task flexibility?

|_| Yes

|_| No

D6. How many co-workers do you have?

|_| fewer than 10 (very small company)

|_| from 10 to 49 (small company)

|_| from 50 to 249 (medium company)

|_| more than 249 (large company)

|_| I work alone

D7. How long have you been at your current job?

|_| less than 1 year

|_| from 1 to 5 years

|_| from 5 to 10 years

|_| more than 10 years

D8. Why have you come to In-Forma Salute? What is your work-related problem?

………
………
………
………
……………………

Section E_ Employed in the private sector

E1. You are:

|_| Self-employed

|_| Employed

E2. If you are self-employed, you are a:

|_| Business owner

|_| Artisan

|_| Freelance professional

E3. If you are employed, you have a:

|_| fixed-term contract

|_| permanent contract

E4. You work as a:

|_| Executive

|_| Manager

|_| Office Worker

|_| Worker

|_| Teacher

|_| Food service worker

|_| Homemaker

|_| Other …………………………………………………..

E5. Does your job have a flexible schedule?

|_| Yes

|_| No

E6. Does your job have work-task flexibility?

|_| Yes

|_| No

E7. How many co-workers do you have?

|_| fewer than 10 (very small company)

|_| from 10 to 49 (small company)

|_| from 50 to 249 (medium company)

|_| more than 249 (large company)

|_| I work alone

E8. How long have you been at your current job?

|_| less than 1 year

|_| from 1 to 5 years

|_| from 5 to 10 years

|_| more than 10 years

E9. Why have you come to In-Forma Salute? What is your work-related problem?

……
……
……
……
……………………

SOCIAL AND WORK QUESTIONNAIRE

PERSONAL DATA

Name and Surname: ……………………………………………………

Age: ……………………………………………………

Contacts (telephone number, email address):……………………………………………………

Address;……………………………………………………

EMPLOYMENT STATUS:

<table>
<tr><td rowspan="3">I am employed, but I am on leave due to cancer</td><td>Are you interested in volunteer activities?
|_| Yes, I am
|_| No, I am not
|_|I already am a volunteer</td></tr>
<tr><td>If so, what is your availability and in which field?
……………………………………………………………………………………………
……………………………………………………………………………………………
…….</td></tr>
<tr><td>Do you think you will need to review/ revise your career path?
……………………………………………………………………………………………
……………………………………………………………………………………………
…….</td></tr>
<tr><td>Unemployed</td><td>|_|I lost my job after receiving my cancer diagnosis

|_|I was already unemployed when I received my cancer diagnosis

|_|I have never worked</td></tr>
</table>

FOR BOTH CASES

**In terms of work and/or training, what do you think your needs are?**	|_| I need training<br><br>|_| I need to find new job opportunities suitable to my current needs<br><br>|_|I need information about the job market
**Do you have a Curriculum Vitae?**	|_| Yes, I do (in this case, bring it to the next appointment with social cooperatives)

	|_| No, I don't

**Do you have permanent or temporary disability?**	|_| Yes, it is certified<br>|_| The certification process is underway<br>|_| No, I don't

**Employment Centre**<br>(Only for unemployed)	|_| I know it exists<br>|_| I am not enrolled<br>|_| I am enrolled<br>|_| I am enrolled in the targeted placement

Provincial Services:

- Have you ever had any kind of contact with a social worker?

|_| Yes, I have
|_| No, I have not

- If so, do you remember his/her name?..

TIDieR
Template for Intervention Description and Replication

The TIDieR (Template for Intervention Description and Replication) Checklist*:

Information to include when describing an intervention and the location of the information

Item number	Item	Where located ** Primary paper (page or appendix number)	Other † (details)
	**BRIEF NAME**		
**1.**	Provide the name or a phrase that describes the intervention.	1	______________
	**WHY**		
**2.**	Describe any rationale, theory, or goal of the elements essential to the intervention.	5 - 8	______________
	**WHAT**		
**3.**	Materials: Describe any physical or informational materials used in the intervention, including those provided to participants or used in intervention delivery or in training of intervention providers. Provide information on where the materials can be accessed (e.g. online appendix, URL).	9 - 15	______________
**4.**	Procedures: Describe each of the procedures, activities, and/or processes used in the intervention, including any enabling or support activities.	10 - 15	______________
	**WHO PROVIDED**		
**5.**	For each category of intervention provider (e.g. psychologist, nursing assistant), describe their expertise, background and any specific training given.	9 - 15	______________
	**HOW**		
**6.**	Describe the modes of delivery (e.g. face-to-face or by some other mechanism, such as internet or telephone) of the intervention and whether it was provided individually or in a group.	10 - 15	______________
	**WHERE**		
**7.**	Describe the type(s) of location(s) where the intervention occurred, including any necessary infrastructure or relevant features.	10 - 15	______________

	**WHEN and HOW MUCH**		
**8.**	Describe the number of times the intervention was delivered and over what period of time including the number of sessions, their schedule, and their duration, intensity or dose.	NA	______________
	**TAILORING**		
**9.**	If the intervention was planned to be personalised, titrated or adapted, then describe what, why, when, and how.	13 - 15	______________
	**MODIFICATIONS**		
**10.‡**	If the intervention was modified during the course of the study, describe the changes (what, why, when, and how).	NA	______________
	**HOW WELL**		
**11.**	Planned: If intervention adherence or fidelity was assessed, describe how and by whom, and if any strategies were used to maintain or improve fidelity, describe them.	NA	______________
**12.‡**	Actual: If intervention adherence or fidelity was assessed, describe the extent to which the intervention was delivered as planned.	NA	______________

** **Authors** - use N/A if an item is not applicable for the intervention being described. **Reviewers** – use '?' if information about the element is not reported/not sufficiently reported.

† If the information is not provided in the primary paper, give details of where this information is available. This may include locations such as a published protocol or other published papers (provide citation details) or a website (provide the URL).

‡ If completing the TIDieR checklist for a protocol, these items are not relevant to the protocol and cannot be described until the study is complete.

* We strongly recommend using this checklist in conjunction with the TIDieR guide (see *BMJ* 2014;348:g1687) which contains an explanation and elaboration for each item.

* The focus of TIDieR is on reporting details of the intervention elements (and where relevant, comparison elements) of a study. Other elements and methodological features of studies are covered by other reporting statements and checklists and have not been duplicated as part of the TIDieR checklist. When a **randomised trial** is being reported, the TIDieR checklist should be used in conjunction with the CONSORT statement (see www.consort-statement.org) as an extension of **Item 5 of the CONSORT 2010 Statement.** When a **clinical trial protocol** is being reported, the TIDieR checklist should be used in conjunction with the SPIRIT statement as an extension of **Item 11 of the SPIRIT 2013 Statement** (see www.spirit-statement.org). For alternate study designs, TIDieR can be used in conjunction with the appropriate checklist for that study design (see www.equator-network.org).
